# ICTV Virus Taxonomy Profile: *Artoviridae*

**DOI:** 10.1099/jgv.0.001292

**Published:** 2019-06-17

**Authors:** Ralf G. Dietzgen, Dàohóng Jiāng, Jens H. Kuhn, Nikos Vasilakis, ICTV Report Consortium

**Affiliations:** ^1^ Queensland Alliance for Agriculture and Food Innovation, The University of Queensland, St. Lucia, Queensland, Australia; ^2^ State Key Laboratory of Agricultural Microbiology, The Provincial Key Lab of Plant Pathology of Húběi Province, College of Plant Science and Technology, Huázhōng Agricultural University, Wǔhàn, PR China; ^3^ Integrated Research Facility at Fort Detrick, National Institute of Allergy and Infectious Diseases, National Institutes of Health, Frederick, MD, USA; ^4^ Center for Biodefense and Emerging Infectious Diseases, Department of Pathology, The University of Texas Medical Branch, Galveston, TX, USA

**Keywords:** ICTV Profile, taxonomy, *Artoviridae*

## Abstract

The family *Artoviridae* was created in 2018 for the established monospecific genus *Peropuvirus* and six new species of invertebrate viruses that had all been discovered by high-throughput sequencing. Artoviruses have negative-sense RNA genomes of about 12 kb and produce enveloped, spherical particles that are 100–130 nm in diameter. Hosts include parasitoid wasps, barnacles, pillworms, woodlice, copepods and odonates. This is a summary of the International Committee on Taxonomy of Viruses (ICTV) Report on the family *Artoviridae*, which is available at www.ictv.global/report/artoviridae.

## Virion

**Fig. 1.**
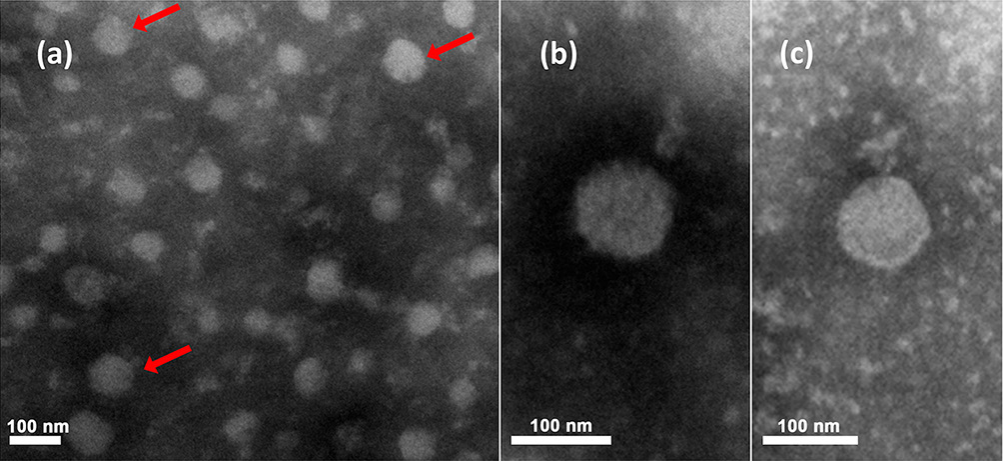
Electron micrographs of purified Pteromalus puparum negative-strand RNA virus 1 particles from follicular cells. (a) Electron micrographs of purified particles. (b) and (c) are magnifications from (a). Red arrows indicated viral particles. Reproduced from [1].

Virions of Pteromalus puparum negative-strand RNA virus 1 are enveloped and spherical [1] with a diameter of 100–130 nm (Table 1, Fig. 1). Virion information is not available for other artoviruses, which are only known from genomic sequence data [2, 3].

**Table 1.** Characteristics of members of the family *Artoviridae*

Typical member:	Pteromalus puparum negative-strand RNA virus 1 (KX431032), species *Pteromalus puparum peropuvirus*, genus *Peropuvirus*
Virion	Enveloped, spherical particles, 100–130 nm in diameter
Genome	Negative-sense, unsegmented RNA of about 12 kb
Replication	Nuclear: the RNA-directed RNA polymerase engages with the ribonucleoprotein complex at the genome 3′-end
Translation	Individual, putatively polyadenylated mRNAs are translated in the cytoplasm
Host range	Barnacles, copepods, odonates, parasitoid wasps, pillworms, woodlice
Taxonomy	Realm *Riboviria*, phylum *Negarnaviricota*, subphylum *Haploviricotina*, class *Monjiviricetes*, order *Mononegavirales*. The genus *Peropuvirus* includes several species

Spherically shaped Pteromalus puparum negative-strand RNA virus 1 particles are present in follicular cells of the ovaries of infected parasitoid wasps (Fig. 1). Similarly, virion-like particles stacked in intracellular vesicles have been observed in cells of the digestive tract. The spherical particles of Pteromalus puparum negative-strand RNA virus 1 are similar to particles produced by nyamiviruses [1, 4].

## Genome

**Fig. 2.**
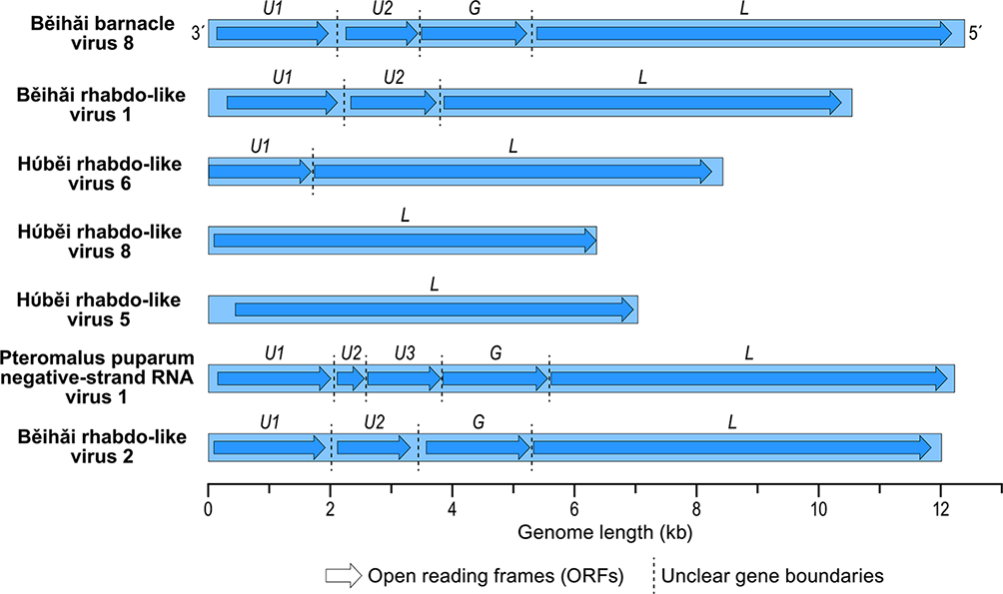
Artovirus genome organization. Some virus genome sequences may be incomplete.

Artovirus negative-sense RNA genomes are of up to 12 kb. All known artoviruses have unsegmented genomes (Fig. 2). The Pteromalus puparum negative-strand RNA virus 1 genome contains five large, independently transcribed, non-overlapping open reading frames (ORFs) encoding three hypothetical proteins of unknown function (U1, U2 and U3), a putative glycoprotein (G) and a large protein (L) encoding an RNA-directed RNA polymerase domain. The genome 3′-leader and 5′-trailer regions have terminal nucleotides that do not exhibit obvious complementarity. Typical conserved transcription initiation and termination motifs are identified upstream and downstream, respectively, of each putative ORF.

## Replication

Knowledge of artovirus replication is limited.

## Pathogenicity

Pteromalus puparum negative-strand RNA virus 1 was originally isolated from a laboratory parasitoid strain of a pteromalid wasp [*Pteromalus puparum* (Linnaeus, 1758)]. The virus is present in various tissues and life stages of the parasitoid and is transmitted vertically through females and males. Virus infection increases adult longevity and impairs several fitness parameters of the wasp, but has no influence on successful parasitism, although it moderates the offspring sex ratio by decreasing female offspring numbers [1].

## Taxonomy

Artoviruses form a family in the haploviricotine order *Mononegavirales* [5]. Within this order, artoviruses are most closely related to members of the families *Bornaviridae*, *Lispiviridae*, *Mymonaviridae* and *Nyamiviridae*. Like most other mononegaviruses, artoviruses (i) have unsegmented, negative-sense RNA genomes; (ii) encode proteins with high sequence identity; (iii) have five conserved motifs (A–E) in the amino acid sequence of their RNA-directed RNA polymerases; (iv) produce enveloped virions; and (v) do not rely on cap-snatching but instead cap their own mRNAs.

## Resources

Full ICTV Report on the family *Artoviridae*: www.ictv.global/report/artoviridae.
